# Beyond Random Splits: Assessing the Generalization of Graph and Vector Models for WT-Structure-Only Drug Resistance Prediction under Protein-Disjoint Evaluation

**DOI:** 10.34133/csbj.0144

**Published:** 2026-06-18

**Authors:** Zongrui Cheng, Haoxin Wu, Dengming Ming

**Affiliations:** College of Biotechnology and Pharmaceutical Engineering, Nanjing Tech University, Nanjing 211816, China.

## Abstract

**Background:** Predicting mutation-induced changes in binding free energy (ΔΔG) is important for understanding drug resistance and prioritizing resistant variants, yet real-world generalization remains unclear. In clinical diagnosis and early-stage drug screening, mutant complex structures are often unavailable. However, many existing methods rely on paired wild-type (WT) and mutant structures and are evaluated under random splits that may permit substantial protein-level train–test overlap. **Methods:** We established a WT-complex-structure-only setting, in which mutant structural coordinates are unavailable but mutation annotations are provided, and conducted a controlled comparison of graph- and vector-based configurations under both random and strict UniProt-based splits. We further analyzed graph context, message passing, representation bias, and dataset noise to identify factors limiting protein-disjoint generalization. **Results:** On MdrDB, random splits yielded apparently moderate performance (Pearson *R* ≈ 0.55), whereas strict UniProt-based evaluation simulating unseen proteins led to a marked drop (Pearson *R* ≈ 0.15), indicating that random splits substantially overestimate generalization. Graph-based modeling retained a weak but nonzero signal relative to the vector baseline, although the differences were limited and not statistically significant. Ablation analyses suggested that full-protein structural context was more useful than local pocket-plus-mutation context, whereas full-protein message passing did not provide a clear additional advantage. ΔESM representations reduced protein-background clustering, but overall strict-split performance remained low. **Conclusions:** WT-only prediction of mutation-induced drug resistance from static structures remains far from solved under realistic protein-disjoint evaluation. Experimental label inconsistency, sparse protein coverage, and missing dynamic structural information may further limit performance, underscoring the need for split-aware evaluation protocols and stronger physical priors.

## Introduction

The emergence of acquired drug resistance represents a formidable bottleneck in precision oncology and infectious disease management, frequently eroding the clinical efficacy of otherwise potent targeted therapies [1]. At the molecular level, such resistance is often driven by nonsynonymous single-nucleotide mutations in target proteins, which can drastically alter the binding affinity of therapeutic ligands. Quantifying this effect via mutation-induced changes in binding free energy (ΔΔG) provides a key thermodynamic metric for assessing resistance potential. Physics-based tools such as FoldX [2], as well as more rigorous alchemical free-energy methods, including free energy perturbation (FEP) and thermodynamic integration (TI), can provide highly accurate estimates of binding free-energy changes. However, their substantial computational cost makes them impractical for large-scale mutational scanning [3,4]. Consequently, data-driven deep learning methods have rapidly emerged as a promising alternative [5,6]. By leveraging large-scale structural and sequence data, these artificial intelligence (AI)-based models aim to efficiently capture complex structure–activity relationships, thereby providing a high-throughput paradigm for predicting drug resistance profiles [7–10].

Recent studies have further demonstrated the broad utility of AI, machine learning, and computational structural biology in drug resistance research. These methods have been applied to antimicrobial resistance prediction, resistance-gene discovery, and genetic-variation analysis, including feature selection from pan-genomic datasets, unitig-centered machine-learning strategies, and computational frameworks for analyzing functional genetic variability [11–14]. Computational structural and evolutionary analyses have also been used to interpret resistance-associated mutations, characterize molecular mechanisms underlying drug resistance, and evaluate split-dependent generalization in mutation-induced resistance prediction [15–17]. Beyond resistance prediction, computational and AI-assisted strategies have also been applied to therapeutic intervention design, including drug repurposing, vaccine engineering, and antimicrobial peptide discovery or optimization [18–21]. Despite these advances, the specific problem of mutation-induced ΔΔG prediction for drug resistance remains challenging, particularly when models must generalize to unseen protein backgrounds under realistic wild-type (WT)-complex-structure-only constraints.

While current deep learning architectures have substantially advanced ΔΔG prediction, state-of-the-art models—including CATH-ddG [22], DDMut [23], and mCSM-lig [24]—remain inherently dependent on the availability of both WT and mutant (MT) structures. This reliance on post-mutational conformations creates a substantial barrier to their application in rapid clinical screening, where MT structures are seldom known a priori*.* This assumption often leads to improved predictive performance, suggesting that MT conformational information contributes substantially to ΔΔG prediction. However, this dependence severely limits the practical applicability of these methods in real-world settings. In clinical diagnosis and early-stage drug screening, only the WT complex structure is typically available. Determining a high-resolution 3-dimensional (3D) structure for every newly emerging clinical mutation through x-ray crystallography or cryo-electron microscopy is not only costly and time-consuming but also often experimentally infeasible. This “scarcity of structural data” makes MT-dependent models difficult to deploy for unseen mutations or large-scale mutational scanning tasks, thereby creating an urgent need for a computational paradigm that can make effective predictions using only the structural context of the WT complex.

Although tools such as AlphaFold can be used to generate MT structures [25], performing structural prediction for a large number of mutations remains computationally expensive for large-scale mutational scanning. Moreover, structures of point MTs generated by AlphaFold and related methods often regress toward the WT conformation, rendering them insufficiently sensitive to subtle side-chain perturbations and mutation-induced structural changes [26,27]. More recently, challenges of protein–ligand cofolding models have also been reported in binding prediction settings. For example, Bret et al. [28] showed that Boltz-2 binding classification can remain insensitive to biologically meaningful binding-site mutations and even target exchange, raising concerns about whether such predictions always reflect physical intermolecular interactions. Therefore, exploring a predictive paradigm that enables millisecond-level inference with zero additional folding cost may have substantial practical value for clinical screening applications. Despite the rapid development of mutation-effect prediction models, a systematic evaluation of their generalization ability in a realistic WT-only prediction setting remains lacking.

To address these practical limitations, this study focuses on mutation-induced ΔΔG prediction in a WT-only setting. Here, the term “WT-only” refers specifically to a WT-complex-structure-only constraint: At inference time, the model has access only to the WT protein–ligand complex structure and the known mutation annotation, whereas any MT complex structures, post-mutational coordinates, relaxed MT conformations, or MT ligand poses are strictly unavailable. Because the amino acid substitution is a known input in practical mutation-effect screening, sequence-derived MT information—such as MT-sequence embeddings or WT–MT embedding differences—is permitted as part of the mutation descriptor, while structural geometry remains exclusively restricted to the WT state. This setting is more realistic for clinical screening and early-stage drug discovery, but also substantially more challenging, as the model must infer mutation-induced perturbations from the WT structural context and sequence-level mutation descriptors. Graph neural networks (GNNs) have recently shown promise in modeling molecular graphs and protein–ligand interactions [29–32]. Building on this capability, graph-based structural representations provide a natural framework for testing whether explicit 3D context can improve WT-only drug resistance prediction in realistic evaluation settings.

Recent studies suggest that dataset partitioning strategies can strongly influence benchmark performance in ΔΔG prediction. In several widely used settings, nonredundancy is enforced at the mutation or sequence level. Yet, the same protein may still appear in both training and test sets through different residue substitutions or structurally related complexes. For example, in DDMut [23], the blind test set is nonredundant at the mutation level, but the same protein can still be shared across splits through mutations at different residue positions. Likewise, although DeepDDG [33] applies sequence-similarity-based filtering, protein- or structure-level redundancy is not explicitly controlled. Such partitioning schemes inherently allow protein-level train–test overlap, leading to in-distribution evaluations that may overestimate out-of-protein generalization. Consistent with this concern, Xie et al. [17] recently showed on the MdrDB dataset that sequence-based models relying on protein language model embeddings suffer substantial performance degradation under strict UniProt-level partitioning. While this recent study provides important evidence that apparent performance can be strongly affected by partitioning strategy, its primary focus was on sequence-based prediction and on using reference information to improve generalization when related measurements are available. In contrast, our study asks a different question: How far can mutation-induced ΔΔG predictions be pushed in a WT-only setting when neither MT conformations nor target-specific reference samples are available at inference time? We therefore position the present work as a split-aware controlled evaluation of a stricter zero-reference structural setting, rather than as an alternative implementation of reference-assisted sequence-based prediction.

Motivated by this gap, we conducted a controlled comparison of vector- and graph-based configurations for mutation-induced drug resistance prediction on the MdrDB dataset under a realistic WT-complex-structure-only setting. Unlike prior studies that primarily exposed the generalization gap in sequence- or vector-based paradigms, this study asks whether explicit 3D structural modeling retains any predictive signal under the out-of-protein generalization gap when neither MT conformations nor target-specific reference samples are available at inference time. Our results show that the relatively strong performance observed under conventional random splits is largely driven by protein-level train–test overlap and does not reflect true cross-protein generalization. Under strict UniProt-based evaluation, all models declined markedly, and graph-based configurations retained only a weak, distance-dependent structural signal rather than solving the protein-disjoint generalization problem.

## Methods

### Dataset

The data on mutation-induced changes in drug binding free energy (ΔΔG) used in this study were obtained from MdrDB (Mutation-induced Drug Resistance DataBase) [34]. MdrDB integrates multiple public data resources, including the Platinum, AIMMS, TKI, RET, KinaseMD, GDSC, and DepMap datasets. Through a unified preprocessing pipeline, it organizes protein names, UniProt IDs, mutation annotations, drug information, and the corresponding binding free-energy changes (ΔΔG) into a standardized sample format. In addition to the core protein–drug–mutation–ΔΔG information, MdrDB provides associated 3D structural data (WT and MT protein–ligand complexes) and structure-related features, thereby providing a structural foundation for constructing and evaluating machine-learning models.

In this study, MdrDB_CoreSet was used as the primary data source. This dataset contains 4,292 protein–ligand mutation records spanning multiple UniProt IDs. We formulated the prediction of mutation-induced changes in binding free energy as a regression task to predict the ΔΔG value for each mutation relative to the wild type (in kcal/mol).

Given that this study focuses on single-point mutations, we further filtered and curated the original dataset as follows:

1. Only samples involving single amino acid substitution mutations were retained;
2. Abnormal complex structures lacking valid protein contacts around the ligand were removed;
3. PDB records with ambiguous structural mapping caused by multichain assemblies or duplicated residue numbering were excluded;
4. Samples with incomplete or unresolvable structural information were discarded.

After the above filtering procedures, a total of 3,125 valid protein–ligand mutation samples were retained for subsequent model training and evaluation, covering proteins from 166 distinct UniProt IDs.

For the experimental design, we constructed both a random split and a strict UniProt-based split. The random split evaluates model fitting under conventional sample-level partitioning. In contrast, the strict UniProt-based split assesses protein-disjoint generalization by ensuring that no proteins are shared between the training and test sets. The detailed sample and unique protein distributions for each cross-validation fold are provided in Table S1. To further quantify the extent of protein-level train–test overlap under random splitting, we also measured the proportion of test samples whose proteins were already present in the training set.

### Protein representation

To characterize the contextual semantic information of protein sequences and the local perturbation features induced by mutations, we constructed multiple residue-level representation schemes, including amino acid identity encoding, embedding-based representations derived from a pretrained protein language model [35,36], and mutation-perturbation representations constructed from embedding differences. Let the WT protein sequence be denoted as S_WT_, and the MT sequence as $S_{\text{MT}}$.

In this study, we used the pretrained protein language model ESM-C (600M parameters) [37] as the feature extractor, denoted by $f_{\mathrm{ESM}}\left(\cdot \right)$. This model is self-supervised and pretrained on large-scale protein sequence data, providing residue-level contextual embeddings. In our framework, the ESM-C parameters were kept frozen and used only for embedding extraction. The sequence encodings are defined as:

$$E^{\mathrm{WT}}=f_{\mathrm{ESM}}\left(S^{\mathrm{WT}}\right),E^{\mathrm{MT}}=f_{\mathrm{ESM}}\left(S^{MT}\right)$$ (1)

where $E^{\mathrm{WT}}={\left\{e_{i}^{\mathrm{WT}}\right\}}_{i=1}^{n}\;$ and $E^{\mathrm{MT}}={\left\{e_{i}^{\mathrm{MT}}\right\}}_{i=1}^{n}$ (n is the sequence length and $e_{i}\in {\mathbb{R}}^{d}\;$ denotes the embedding vector of the ith residue).

1. One-hot representation

A 20D one-hot vector was used to encode the amino acid identity of each residue:

$$x_{i}\in {\mathbb{R}}^{20}$$ (2)

where i denotes the residue index. This representation contains only amino acid category information and serves as a basic sequence feature.

2. ESM embedding representation

ESM-C was used to extract residue-level embeddings for the WT and MT sequences, denoted as $E^{\mathrm{WT}}$and $E^{\mathrm{MT}}$, respectively, to incorporate evolutionary and contextual semantic information.

3. ΔESM differential representation

Because ΔΔG reflects the energetic difference relative to the WT state, we further constructed a residue-level differential representation based on ESM-C embeddings:

$$E^{\Delta }=E^{\mathrm{WT}}-E^{\mathrm{MT}}$$ (3)

where $E^{\Delta }={\left\{e_{i}^{\Delta }\right\}}_{i=1}^{n}$ and $e_{i}^{\Delta }\in {\mathbb{R}}^{d}$. This representation is used to explicitly characterize mutation-induced perturbations at the representation level, thereby aligning the feature formulation with the relative definition of ΔΔG.

### Mutation-site indicator encoding

In mutation-induced ΔΔG prediction tasks, the perturbation is typically confined to a single amino acid position. However, full-protein sequence- or structure-based representations do not, by default, explicitly distinguish the mutated site from all other residues, which may weaken the model’s ability to focus on the functionally critical position. To address this issue, we introduced a mutation-site indicator vector. Let $r_{i}$ denote the residue position associated with the ith node, and let $r_{\mathrm{mut}}$ denote the mutated residue position for the given sample. We define:

$$m_{i}=\left\{\begin{array}{cc} 1, & r_{i}=r_{\mathrm{mut}}\\ 0, & r_{i}\neq r_{\mathrm{mut}} \end{array}\right.$$ (4)

where $m_{i}$ is the mutation-site indicator variable for the ith node. This indicator is appended to the protein node features as an additional input channel to mark the unique mutated site explicitly.

In addition, we constructed a 20D one-hot difference vector for the amino acid substitution and an 8D difference vector for physicochemical properties:

$$\mathrm{\Delta o}=o^{\mathrm{MT}}-o^{\mathrm{WT}},\mathrm{\Delta p}=p^{\mathrm{MT}}-p^{\mathrm{WT}}.$$ (5)

Within the model, these difference vectors are first projected into the hidden space and then injected exclusively into the mutated node’s representation under the gating effect of $m_{i}$. Furthermore, the original difference vectors are concatenated with the global features at the regression stage to provide sample-level mutational attribute information.

### Ligand representation

To compare the effects of different representation paradigms on ΔΔG prediction, we adopted 2 types of ligand representations in this study: one based on vectorized features (nongraph representation) and the other based on an atom-level graph representation (graph-based model). Both representations were derived by parsing the ligand 3D structure files in SDF format.

#### Vectorized ligand representation

Under the vectorized setting, each ligand was encoded using topology-based Morgan fingerprints, also known as extended-connectivity fingerprints (ECFPs) [38], yielding a fixed-length binary vector:

$$f_{\mathrm{lig}}=\text{ECFP}\left(\text{ligand};\;R,\;B\right)\in {\left\{0,\;1\right\}}^{B},$$ (6)

where R denotes the fingerprint radius (set to R=2 in this study) and B denotes the fingerprint bit length (set to B=1,024 in this study). This vector was used as the ligand-side input feature and combined with the protein-side representation for downstream regression.

#### Graph-based ligand representation

In the graph-based setting, each ligand was represented as an atom-level graph $G_{\mathrm{lig}}=\left(V_{\mathrm{lig}},\;E_{\text{bond}}\right)$, where $V_{\mathrm{lig}}$ denotes the set of ligand atoms and $E_{\text{bond}}$ denotes the set of covalent bond connections. For each atom node, a 40D atomic feature vector was constructed, and for each bond edge, a 20D edge feature vector was defined, incorporating information such as bond type and bond length. To ensure consistency among atom indexing, atomic coordinates, and bond connectivity, the ligand molecules were standardized, and explicit hydrogen atoms were removed.

Within the graph-based model, the ligand graph was further connected to the protein residue graph via cross-type edges defined by spatial contact relationships, thereby enabling the explicit representation of protein–ligand interactions. The details of the corresponding graph construction and the heterogeneous GNN architecture are described in detail in the following sections.

### Protein feature construction schemes

Importantly, the designation “WT-only” in this study denotes the absence of MT structural coordinates rather than the exclusion of MT sequence annotations. The MT residue identity is part of the known mutational input, and MT-sequence-derived embeddings are employed strictly as sequence-level mutation descriptors in selected modes, such as mode 4. No MT protein–ligand complex structure, simulated MT coordinates, or post-mutation ligand geometries are introduced in any of the evaluation modes.

To assess the effects of different representation strategies on ΔΔG prediction performance, we defined 5 input feature configurations (modes 0 to 4). All modes were evaluated under a unified training framework to quantify the contributions of graph-based modeling and different protein embedding strategies. These configurations were designed to disentangle the effects of explicit structural modeling and different mutation-aware protein representations under the same WT-only prediction setting.

#### Mode 0: Vector baseline model

In this mode, no protein–ligand graph structure was constructed. Instead, regression was performed using sample-level vector representations. The protein feature was defined as the global mean-pooled representation of sequence-level embeddings, concatenated with the mutation difference vectors and the ligand molecular fingerprint (ECFP), forming the model input. This mode was used to evaluate baseline performance in the absence of explicit structural information.

#### Mode 1: Graph + One-hot

In this mode, a protein–ligand heterogeneous graph was constructed, and protein nodes were represented solely by amino acid one-hot encodings. Mutation-related difference vectors were injected into the mutated residue node via a mutation-aware mechanism and subsequently concatenated with global features at the readout stage. This mode was used to evaluate the representational capacity of basic residue identity information within a graph-based framework.

#### Mode 2: Graph + One-hot + WT-ESM

Based on mode 1, residue-level ESM embeddings of the WT sequence were incorporated to enhance the representation of evolutionary and contextual semantic information.

#### Mode 3: Graph + One-hot + ΔESM

In this mode, residue-level differential embeddings (ΔESM) were used in place of absolute embedding representations, enabling explicit modeling of mutation-induced perturbation effects.

#### Mode 4: Graph + One-hot + WT-ESM + MT-ESM

In this mode, both WT and MT residue-level embeddings were retained, allowing the model to learn their relational differences directly within the graph-based architecture.

### Graph construction

The graph construction pipeline was partially adapted from the open-source GEMS implementation [32]. Still, the final representation was formulated as a heterogeneous graph to distinguish protein residues, ligand atoms, and their interaction relations [39–41]. Each protein–ligand complex was represented as a heterogeneous graph.

$$G=\left(V,\;E\right),$$ (7)

where the node set V consists of 2 types of entities: ligand heavy-atom nodes and protein residue nodes.

**Fig. 1.**
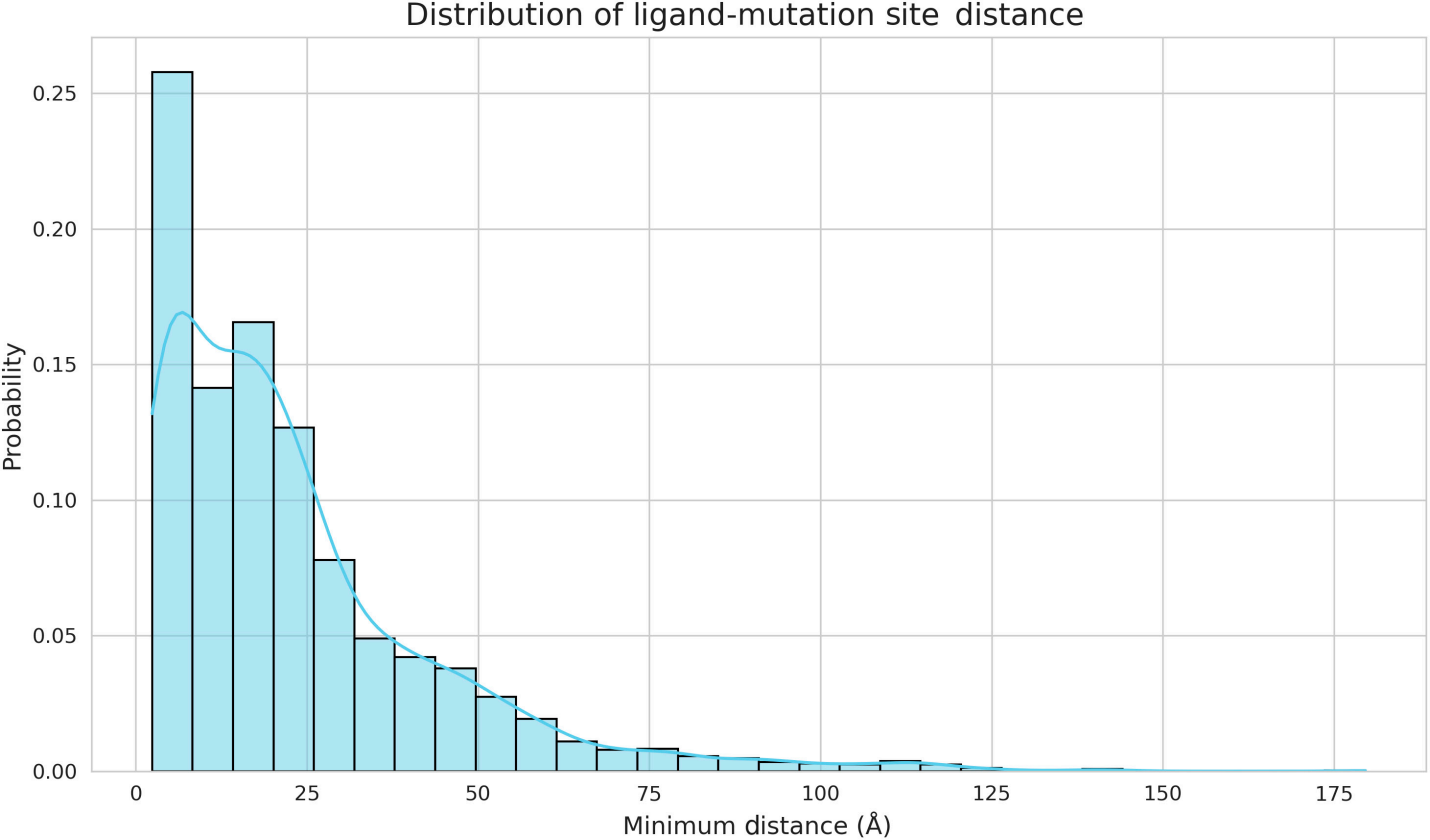
Distribution of distances between the ligand and mutation sites.

Unlike pocket-only representations, we retained the full protein structure as graph input. This choice was motivated by the long-tailed mutation–ligand distance distribution in MdrDB (Fig. 1), where many mutation sites are located far from the ligand-binding pocket. A pocket-only graph would remove the mutated residue and its local structural context for these distal cases. Nevertheless, because static WT structures cannot directly capture mutation-induced conformational dynamics, the usefulness of full-protein context was evaluated empirically through the ablation and stratified analyses described in the “Graph-based models retain a weak, distance-dependent structural signal under strict evaluation” section.

Ligands were represented by heavy atoms with coordinates obtained from SDF conformations. Ligand node features encoded basic chemical attributes, including atom type, ring membership, hybridization, formal charge, aromaticity, atomic mass, hydrogen count, degree, and chirality. Proteins were represented at the residue level. Residue coordinates were assigned using the Cα atom when available; otherwise, the geometric center of the residue was used. The basic residue feature was a 20D amino acid one-hot encoding, with residue-level ESM features appended according to the feature construction scheme described in the previous section.

The edge set included ligand–ligand, protein–protein, and protein–ligand relations. Ligand–ligand edges were constructed from RDKit-defined covalent bonds [42]. Protein–protein edges included sequential adjacency edges between neighboring residues on the same chain and spatial *k*-nearest-neighbor edges based on inter-residue distances, with *k* = 16. Protein–ligand contact edges were added when the distance between a ligand atom and any atom of a protein residue was below 8 Å. All relation edges were instantiated bidirectionally, and self-loop edges were added through a dedicated self-relation. Edge features were uniformly encoded as 20D vectors containing relation-type indicators and geometric distance terms normalized by 10 Å.

A virtual master node was further introduced to provide a graph-level contextual representation across node types. Its coordinate was placed at the geometric center of the protein and ligand nodes, and it was connected to all ligand atom nodes and protein residue nodes. During readout, the master-node representation was incorporated into the final feature fusion.

### Model architecture

**Fig. 2.**
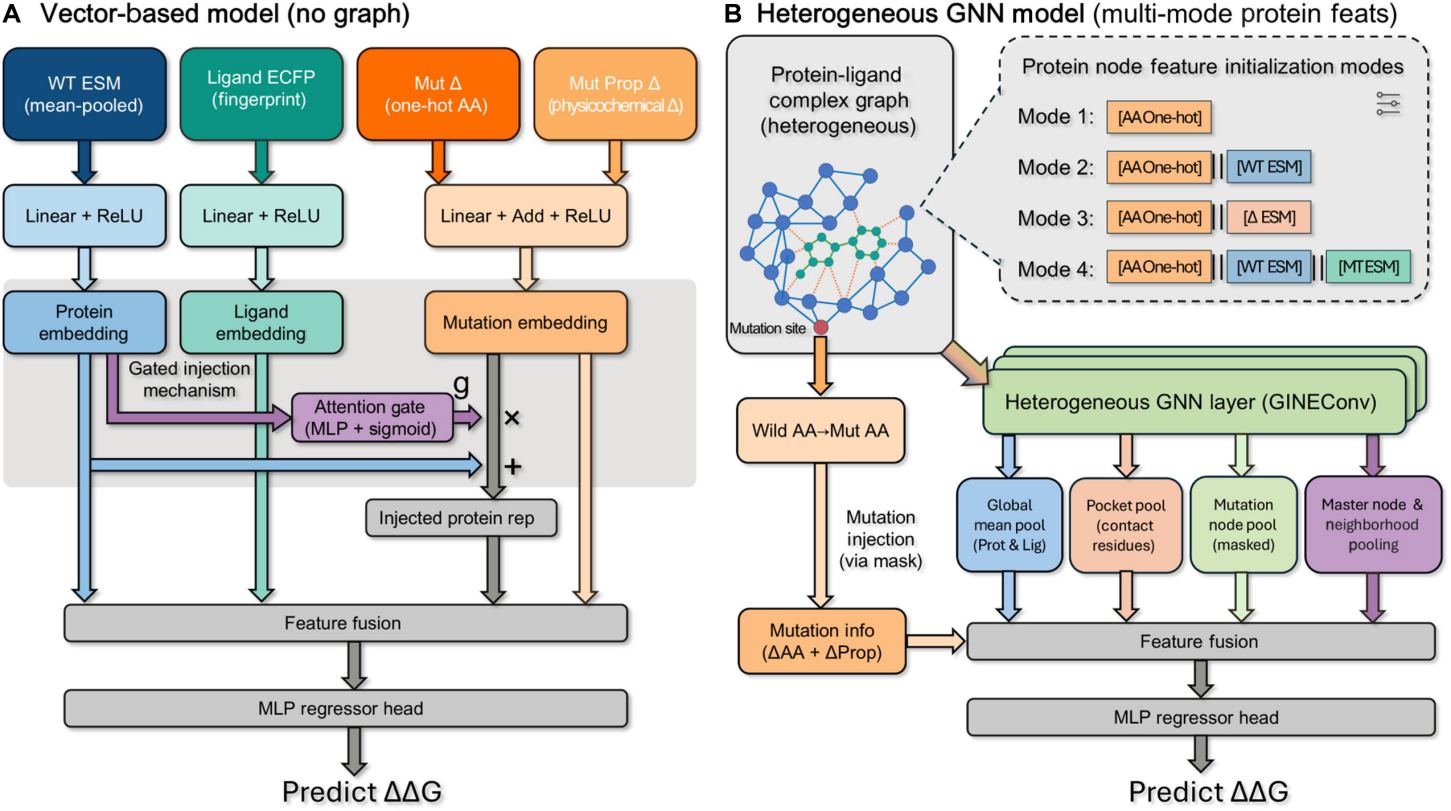
Overview of the evaluated vector and graph configurations. (A) Architecture of the vector-based baseline model. (B) Architecture of the heterogeneous graph neural network (GNN) model incorporating multiple protein feature modes.

Figure 2 summarizes the vector and graph configurations evaluated in this study. Rather than aiming to introduce a standalone predictive architecture, these models were implemented as controlled configurations to assess how different WT-complex-structure-only representations behave under random and protein-disjoint evaluation.

The vector baseline represents each sample using the WT protein sequence embedding, the ligand fingerprint, and mutation descriptors. The mutation information includes the amino acid identity difference and physicochemical property difference, which are projected through separate linear layers and fused into a mutation embedding. This mutation embedding is injected into the global protein representation through a gated modulation mechanism:

$$g=\sigma \left(\mathrm{MLP}\left(\left[h_{p}∥h_{m}\right]\right)\right),{\tilde{h}}_{p}=h_{p}+g⊙h_{m}$$ (8)

Here, σ denotes the sigmoid function and ⊙ denotes element-wise multiplication. The original protein representation, ligand representation, mutation embedding, and mutation-conditioned protein representation are then concatenated and passed to a multilayer perceptron (MLP) regressor.

In the heterogeneous graph model, each protein–ligand complex is represented as a graph containing protein residue nodes, ligand atom nodes, and a virtual master node. Node features are first projected into a shared hidden space, followed by edge-aware heterogeneous message passing using HeteroConv and GINEConv layers [43]. The update rule for a single layer is written as:

$$h_{i}^{\left(k+1\right)}=\phi^{\left(k\right)}\left(h_{i}^{\left(k\right)},\sum_{j\in N\left(i\right)}\psi^{\left(k\right)}\left(h_{i}^{\left(k\right)},\;h_{j}^{\left(k\right)},\;e_{\mathrm{ij}}\right)\right)$$ (9)

Here, $e_{\mathrm{ij}}$ denotes the edge feature, ψ denotes the message function that jointly incorporates node and edge attributes, and ϕ denotes a nonlinear mapping. Through multi-layer propagation, the model progressively integrates local geometric topology and cross-type interaction information.

Based on the mutation-site indicator mask, the fused mutation embedding is selectively injected into the corresponding residue node representation together with an independent positional mask bias:

$$h_{i}^{\prime }=h_{i}+{\text{Linear}}_{\text{mask}}\left(m_{i}\right)+m_{i}\cdot u$$ (10)

The mutated site therefore receives both mutation descriptor information and an explicit positional marker.

At readout, the model combines global and local graph representations, including globally pooled protein features, pooled ligand features, the virtual master-node representation, the mutated-residue representation, and a binding-pocket representation derived from protein–ligand contact edges.

To incorporate local spatial information around the mutation site, we used Gaussian distance-weighted mutation-neighborhood pooling. Let *d* denote the Euclidean distance between a residue and the mutated site. The radial weight is defined as:

$$w_{j}\propto \text{exp}\left(-\frac{d_{j}^{2}}{2\sigma^{2}}\right)$$ (11)

This produces a mutation-neighborhood representation in which residues closer to the mutation site receive larger weights.

Finally, the graph-level representations are concatenated with the original mutation descriptors through a feature-bypass connection:

$$z=\left[g_{\text{prot}}∥g_{\mathrm{lig}}∥g_{\text{master}}∥g_{\text{pocket}}∥g_{\mathrm{mut}}∥g_{\mathrm{nei}}∥\mathrm{\Delta AA}∥\Delta \text{Prop}\right]$$ (12)

The fused representation is passed to an MLP regressor to predict ΔΔG. All models are trained using the Huber loss to improve robustness to noisy experimental labels.

### Training strategy

Model performance was evaluated using 5-fold cross-validation. In each fold, approximately 20% of the samples were held out as the test set. In comparison, the remaining 80% were further divided into training and validation sets for model selection and early stopping. Accordingly, the overall data split was approximately 0.68/0.12/0.20 for the training, validation, and test sets, respectively.

To improve robustness to outlier samples, model training was optimized using the Huber loss [44]. To stabilize the training process, the ΔΔG labels in the training set were standardized via *z*-score normalization, and predictions were rescaled to the original scale during validation and testing before metric calculation. Model parameters were updated using the AdamW optimizer [45], and an early stopping strategy was adopted to prevent overfitting. All experiments were conducted under fixed random seeds to ensure reproducibility.

Model performance was assessed using mean absolute error (MAE), root mean square error (RMSE), the Pearson correlation coefficient, and the Spearman correlation coefficient. The final results are reported as the average test-set performance across the 5 folds.

All deep learning models in this study were implemented using the PyTorch and PyTorch Geometric frameworks [46,47]. For the graph-based models (modes 1 to 4), we adopted a heterogeneous GNN architecture comprising 2 message-passing layers (HeteroConv combined with GINEConv), with a hidden dimension of 128 and a dropout rate of 0.35; interlayer feature aggregation was performed using sum reduction. The vector-based baseline model (mode 0) was implemented as a MLP with a hidden dimension of 512 and a dropout rate of 0.25. All models were optimized using AdamW. To mitigate overfitting, an early stopping mechanism was employed with a patience of 20 epochs. Detailed hyperparameter settings and training configurations are provided in Table S2.

The core hyperparameters, including the k-nearest-neighbor (k-NN) density, contact distance threshold, and Gaussian width σ, were empirically evaluated using mode 3. A detailed sensitivity analysis and an ablation study for the Gaussian pooling mechanism are provided in Table S3.

### Ablation analyses

To examine the contributions of full-protein structural context and non-self spatial message passing, we performed 2 additional mode 3 ablations under the strict UniProt-based split. In the first ablation, termed mode 3-NoMP, the same full-protein nodes, ligand nodes, input features, readout modules, and learnable parameter count as mode 3 were retained, but all non-self message-passing edges were removed. This control was designed to isolate whether the weak numerical signal of mode 3 was attributable to neighbor aggregation through the spatial graph topology. In the second ablation, termed mode 3-PocketMut, we constructed a reduced-context graph by retaining ligand atoms, protein residues within 15 Å of the ligand, and residues within 10 Å of the mutation site. This variant was designed to test whether the ligand-proximal region and mutation-site local neighborhood were sufficient to replace the full-protein structural context. All ablation experiments used the same 5-fold strict UniProt-based split, training protocol, optimization strategy, and hyperparameters as the main mode 3 experiment, without additional hyperparameter tuning.

## Results and Discussion

### Random splits substantially overestimate model generalization

Across all evaluated configurations, the most prominent finding was a substantial split-dependent generalization gap. Under conventional random splits, models achieved apparently moderate predictive performance, with Pearson correlations ranging from approximately 0.48 to 0.55. By contrast, when evaluation was performed under a strict UniProt-based split designed to simulate prediction on unseen proteins, performance declined sharply across all models, with Pearson correlations dropping to approximately 0.04 to 0.15. These results indicate that conventional random splits can substantially overestimate cross-protein generalization when predicting WT-only mutation-induced drug resistance.

Among them, modes 0 and 2 showed the best performance, reaching Pearson correlations of 0.5545 ± 0.0581 and 0.5549 ± 0.0271, respectively, with both models yielding RMSE values close to 1.0. By contrast, mode 1, which used only amino acid one-hot representations, exhibited the weakest performance (Pearson = 0.4873 ± 0.0156, RMSE = 1.0475 ± 0.0392), indicating that residue identity information alone is insufficient to adequately capture the effects of mutations on binding free energy.

The incorporation of pretrained protein language model embeddings (modes 2 to 4) consistently improved performance relative to the one-hot-only representation (mode 1), indicating that sequence contextual information makes a substantial contribution to ΔΔG prediction. However, the performance differences among the different ESM-based representation strategies were relatively limited. Neither the differential embedding representation (mode 3) nor the WT + MT concatenated representation (mode 4) showed a clear advantage over the single WT embedding (mode 2). This may suggest that, under the random split setting, substantial protein-level similarity exists between the training and test samples, allowing the model to obtain sufficiently informative signals even from relatively simple feature formulations.

Overall, model performance under random splitting was stable, with relatively small standard deviations across folds, indicating good training consistency (Table 1).

**Table 1.** Performance metrics under the random split setting (kcal/mol)

Mode	Pearson	Spearman	MAE	RMSE
Mode 0	0.5545 ± 0.0581	0.5411 ± 0.0399	0.7121 ± 0.0158	0.9941 ± 0.0303
Mode 1	0.4873 ± 0.0156	0.4554 ± 0.0290	0.7567 ± 0.0359	1.0475 ± 0.0392
Mode 2	0.5549 ± 0.0271	0.5255 ± 0.0411	0.7223 ± 0.0283	0.9971 ± 0.0525
Mode 3	0.5194 ± 0.0385	0.4896 ± 0.0275	0.7471 ± 0.0340	1.0281 ± 0.0525
Mode 4	0.5388 ± 0.0438	0.5076 ± 0.0500	0.7356 ± 0.0387	1.0130 ± 0.0574

Under the strict UniProt-based split protocol, the performance of all models declined markedly (Table 2). The Pearson correlation coefficients ranged from 0.04 to 0.15 overall, while the RMSE increased to approximately 1.25 to 1.31.

**Table 2.** Performance metrics under the strict UniProt-based split setting (kcal/mol)

Mode	Pearson	Spearman	MAE	RMSE
Mode 0	0.0439 ± 0.0774	0.0436 ± 0.0830	0.9308 ± 0.1600	1.3101 ± 0.3731
Mode 1	0.0897 ± 0.1472	0.1418 ± 0.0973	0.8679 ± 0.0588	1.2558 ± 0.2423
Mode 2	0.1001 ± 0.1108	0.0906 ± 0.1160	0.8645 ± 0.0383	1.2536 ± 0.2421
Mode 3	0.1526 ± 0.1481	0.0863 ± 0.1724	0.8838 ± 0.0907	1.2539 ± 0.2749
Mode 4	0.1033 ± 0.1163	0.0960 ± 0.1336	0.8535 ± 0.0405	1.2458 ± 0.2357

Taking mode 0 as an example, its Pearson correlation dropped from 0.5545 under the random split to 0.0439 under the UniProt-based split, indicating that the predictive correlation was nearly lost. A similar trend was observed across the other modes. This finding suggests that, under random splitting, the models may benefit from protein-level similarity between samples. In contrast, strict UniProt-based partitioning leads to a substantial decline in generalization performance on unseen proteins.

Among the different modes, mode 3 achieved the highest mean Pearson correlation (0.1526 ± 0.1481), whereas mode 4 showed slightly lower mean MAE and RMSE. However, the differences among modes were small relative to the fold-to-fold variability under the strict UniProt-based split. To assess whether these numerical differences were statistically supported, we performed paired fold-level Wilcoxon signed-rank tests using the same 5-fold evaluation protocol. The tests did not indicate statistically significant differences between mode 3 and the other modes across Pearson, Spearman, MAE, or RMSE (Table S4). Therefore, these results should be interpreted as limited numerical trends rather than conclusive evidence that any single mode statistically outperforms the alternatives.

Overall, these results indicate that evaluation under strict UniProt-based partitioning provides a substantially more stringent assessment of WT-only generalization than conventional random splitting. This split-dependent performance gap is consistent with the underlying data composition: More than 95% of test samples in the random split were associated with proteins already seen during training, whereas the strict UniProt-based split enforced 0% protein overlap.

### WT-only prediction remains challenging under strict UniProt-based evaluation

**Fig. 3.**
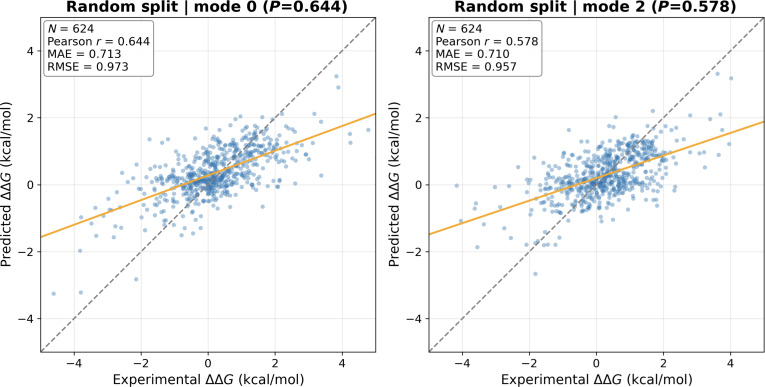
Predicted versus experimental ΔΔG under the random split setting.

To illustrate representative prediction distributions, we visualized the test-set predictions from the best-performing model in a selected fold, with model selection based on validation-set performance in that fold. The ideal diagonal line and the fitted linear regression line were overlaid for reference (Fig. 3). The primary conclusions, however, are based on the mean ± standard deviation across all 5 folds.

Under the random split setting, the models exhibited an overall favorable fit, with predicted values clustered near the diagonal, indicating reasonable agreement with the experimental measurements. After incorporating graph-structural information and protein embeddings (mode 2), performance remained comparable to the vector-based baseline, with the highest Pearson correlation reaching 0.578. This suggests that when training and test samples share substantial protein-level background information, the models can already fit the data effectively by exploiting local statistical regularities, and more complex structural modeling confers little additional performance benefit in this setting.

**Fig. 4.**
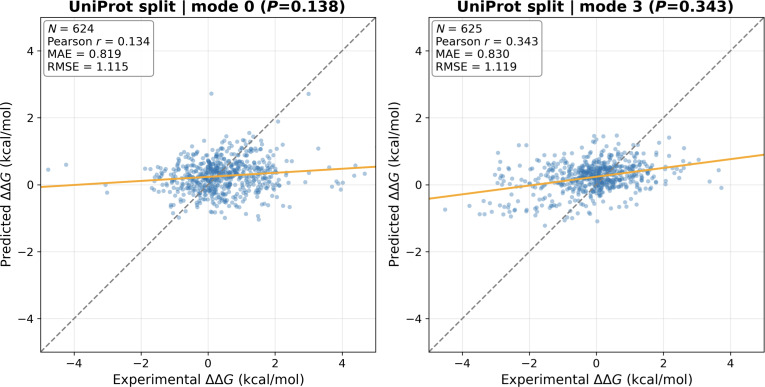
Predicted versus experimental ΔΔG under the UniProt-based split setting.

By contrast, under the strict UniProt-based split, model generalization deteriorated substantially. The Pearson correlation of mode 0 decreased markedly, and the predicted range became noticeably compressed, with a flatter regression slope, indicating that the model struggled to maintain stable predictive performance on previously unseen protein backgrounds. This observation further supports the concern that random splitting may overestimate model performance. Notably, however, under this more stringent setting, the graph-based model (mode 3) showed a less compressed prediction distribution than the vector-based model in this representative fold. Its prediction distribution was visibly more dispersed than that of mode 0, although the overall correlation remained weak and the variance was larger. Nevertheless, these observations suggest that explicit modeling of protein–ligand structural relationships may retain weak structural signal under strict evaluation, although the fold-level differences were not statistically conclusive (Fig. 4).

Taken together, the random split primarily reflects the models’ ability to fit, whereas the UniProt-based split more faithfully evaluates generalization in realistic cross-protein prediction scenarios. Under this stringent setting, all models remain weak, highlighting the difficulty of WT-only ΔΔG prediction on genuinely unseen proteins.

### Graph-based models retain a weak, distance-dependent structural signal under strict evaluation

To quantify the extent of protein-level train–test overlap under different partitioning strategies, we examined whether proteins in the test set were already present in the training set. Under the random split protocol, more than 95% of the test samples corresponded to proteins already seen during training, indicating extensive protein-level overlap between the 2 sets. In contrast, the strict UniProt-based split enforced 0% protein overlap, ensuring that all test proteins were completely unseen during training. This difference in data distribution provides a direct explanation for the substantial performance gap observed between the 2 evaluation settings.

#### Strict-split performance collapse and limited graph-model trends

Within this more realistic protein-disjoint setting, mode 3 achieved a numerically higher mean Pearson correlation than the vector baseline (0.1526 versus 0.0439). However, this difference was modest and not statistically conclusive across folds. Therefore, the result should be interpreted as a limited numerical trend rather than definitive evidence of graph-model superiority. Together with the following stratified analyses, this suggests that explicit structural context may retain a weak, regime-dependent predictive signal under strict cross-protein evaluation, rather than uniformly mitigating the generalization collapse.

#### Mechanistic dissection via graph-context and topology ablations

To further clarify what the graph model contributes under the strict protein-disjoint setting, we performed 2 additional ablations of mode 3 (Table 3). These ablations were designed to separately test the role of full-protein structural context and non-self spatial message passing. First, we constructed a reduced-context graph, termed mode 3-PocketMut, which retained ligand atoms, protein residues within 15 Å of the ligand, and residues within 10 Å of the mutation site. Compared with the full-protein mode 3 graph, this reduced-context variant showed a clear performance decrease, with Pearson decreasing from 0.1526 ± 0.1481 to 0.0552 ± 0.0898 and MAE increasing from 0.8838 ± 0.0907 to 0.9477 ± 0.0765. This result suggests that the ligand-proximal region and mutation-site local neighborhood alone are insufficient to replace the broader full-protein structural context.

**Table 3.** Graph-context and topology ablation of mode 3 under the strict UniProt-based split. Mode 3-NoMP retained the same full-protein nodes, input features, readout modules, and learnable parameter count as mode 3, but removed all non-self message-passing edges. Mode 3-PocketMut retained ligand atoms, protein residues within 15 Å of the ligand, and residues within 10 Å of the mutation site. All experiments used the same 5-fold strict UniProt-based split and training protocol.

Configuration	Nodes retained	Spatial message passing	Pearson R	Spearman S	MAE (kcal/mol)	RMSE (kcal/mol)
Mode 3 (full graph)	Full protein + Ligand	Enabled	0.1526 ± 0.1481	0.0863 ± 0.1724	0.8838 ± 0.0907	1.2539 ± 0.2749
Mode 3 (topology-ablated)	Full protein + Ligand	Disabled (non-self edges)	0.1711 ± 0.1203	0.1230 ± 0.0827	0.9108 ± 0.1534	1.2737 ± 0.3754
Mode 3 (local-context)	Pocket + Mut-Neighborhood	Enabled	0.0552 ± 0.0898	0.0485 ± 0.0940	0.9477 ± 0.0765	1.3389 ± 0.2912

Second, we introduced a topology-ablated control, termed mode 3-NoMP, which retained the same full-protein nodes, input features, readout modules, and learnable parameter count as mode 3, but removed all non-self message-passing edges. Mode 3-NoMP achieved numerically higher Pearson and Spearman correlations than the intact mode 3 model, but slightly worse MAE and RMSE. Therefore, this result should not be interpreted as a clear overall improvement. Instead, it indicates that full-protein neighbor message passing itself does not consistently provide an additional advantage under strict protein-disjoint evaluation. Together, these ablations suggest that retaining the full-protein structural context is beneficial, whereas global neighbor aggregation across the entire protein may introduce noisy or nonrobust signals in the current WT-only static-structure setting.

#### Multi-dimensional stratification and failure-mode analysis

**Fig. 5.**
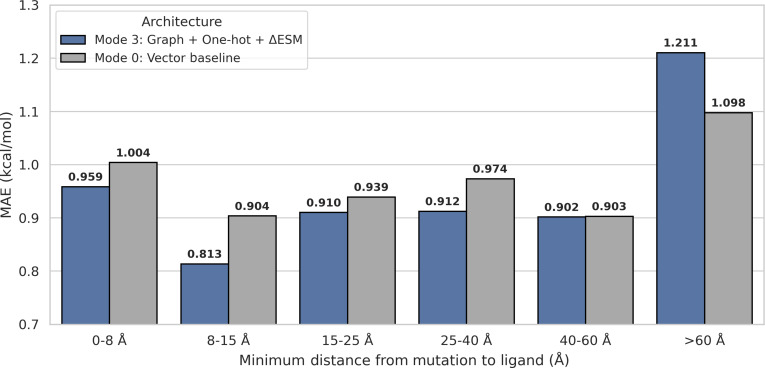
Distance-stratified MAE analysis comparing modes 0 and 3 under the strict UniProt-based split.

We next examined whether this limited structural signal varied across spatial regimes by stratifying the test-set MAE by the minimum distance between the mutation site and the ligand, using the strict UniProt-based split (Fig. 5).

The minimum distance between the mutation site and the ligand stratified test-set MAE. The number of graph instances in each bin was 0 to 8 Å, *n* = 791; 8 to 15 Å, *n* = 538; 15 to 25 Å, *n* = 784; 25 to 40 Å, *n* = 509; 40 to 60 Å, *n* = 322; and >60 Å, *n* = 181.

Mode 3 showed lower MAE than did mode 0 for mutations within 0 to 40 Å of the ligand, with the largest improvement observed in the 8- to 15-Å region. However, this advantage disappeared in the 40- to 60-Å range and reversed beyond 60 Å. These results indicate that graph-based structural modeling appears to provide distance-regime-dependent benefits, primarily for local-to-intermediate mutations in which geometric context remains informative. In contrast, extremely distal mutations remain difficult to predict from static WT structures alone.

In addition to spatial distance, we stratified prediction errors by the magnitude of experimental ∣ΔΔG∣ (Table 4). Mode 3 substantially reduced MAE in the low-magnitude group $\left(|\Delta \Delta G|<0.5\right.$kcal/mol; 0.394 versus 0.494), whereas the 2 models performed similarly for medium-magnitude mutations $\left(0.5\leq |\Delta \Delta G|<1.5\right.$ kcal/mol; 0.875 versus 0.863). For high-magnitude mutations $\left(|\Delta \Delta G|\geq 1.5\right.$kcal/mol), both models showed large errors above 2 kcal/mol, indicating that large-effect mutations remain a major failure mode under the WT-only static-structure setting.

**Table 4.** MAE stratified by experimental |ΔΔG| magnitude under the strict UniProt-based split

|ΔΔG| group	*n*	Mode 0 MAE	Mode 3 MAE
<0.5 kcal/mol	1,247	0.494	0.395
0.5–1.5 kcal/mol	1,285	0.863	0.876
≥1.5 kcal/mol	593	2.153	2.136

Finally, we examined whether prediction errors varied across protein backgrounds by performing target-level stratification based on the PROTEIN_NAME annotation. Because fragmented InterPro identifiers represent the PROTEIN_FAMILY field in MdrDB and contain a substantial fraction of “Unknown” entries, PROTEIN_NAME was used as a more interpretable grouping variable. Major targets with at least 50 test samples were reported individually, whereas smaller targets were merged into an “Other targets” group (Table S5). This analysis showed clear target-dependent heterogeneity: Mode 3 achieved lower MAE than mode 0 in 9 of the 14 major targets, including epidermal growth factor receptor (EGFR), p53, B-raf, Bruton’s tyrosine kinase (BTK), anaplastic lymphoma kinase (ALK), insulin-like growth factor 1 receptor (IGF1R), and DNA topoisomerase 1. However, this advantage was not universal. Mode 3 performed worse for ataxia telangiectasia mutated (ATM) and mechanistic target of rapamycin (mTOR), both of which had large mean mutation–ligand distances greater than 56 Å. This observation is consistent with the distance-stratified analysis and further supports the conclusion that graph-based structural modeling appears to provide target- and distance-dependent benefits rather than uniform improvements across all protein backgrounds.

Together, these stratified analyses suggest that the strict-split performance collapse is not uniform across the dataset. Instead, model behavior depends on the spatial regime, effect size, and target background, highlighting the need for regime-aware or protein-context-aware modeling strategies in future WT-only ΔΔG prediction.

#### Contextual comparison with prior split-aware studies

Our observations are broadly consistent with the recent MdrDB-based study by Xie et al. [17], whose sequence- and vector-based models also used protein language model embeddings and ligand fingerprints. Notably, the behavior of our mode 0 vector baseline—constructed from global protein embeddings, mutation descriptors, and ligand fingerprints—closely resembles the modeling paradigm adopted in such sequence/vector approaches. Under the random split, our mode 0 achieved a mean Pearson correlation of 0.5545, comparable to the ~0.54 correlation reported for the better-performing deep neural network (DNN)/gated recurrent unit (GRU) baselines in that study. Under the strict UniProt-based split, however, our mode 0 dropped sharply to 0.0439, indicating that predictive correlation was nearly lost. To facilitate broader contextualization, representative results from Xie et al. [17] and our models are summarized in Table 5.

**Table 5.** Approximate contextual comparison between representative MdrDB-based results from Xie et al. and our WT-complex-structure-only models. This table is intended as an approximate contextual comparison rather than a direct head-to-head benchmark. The reported results differ in input information, model paradigm, preprocessing, split construction, and evaluation protocol; therefore, direct numerical superiority should not be inferred from these cross-paper values.

Study/architecture	Representation type	Random Pearson	UniProt Pearson
Xie et al. [17]			
RF	WT/MT ESM + ECFP	0.49 ± 0.09	0.06 ± 0.10
DNN	WT/MT ESM + ECFP	0.54 ± 0.07	−0.01 ± 0.10
Our study			
Mode 0	WT-ESM + ECFP	0.55 ± 0.06	0.04 ± 0.08
Mode 3	Graph + One-hot + ΔESM	0.52 ± 0.04	0.15 ± 0.15

Several methodological differences should be noted when interpreting this comparison. First, the input settings differ: Our models operate in a WT-complex-structure-only and zero-reference inference setting, without access to MT structural geometries, post-mutational ligand poses, or target-specific reference measurements at inference time, whereas Xie et al. primarily evaluated sequence/vector-based models using WT/MT ESM embeddings and ligand fingerprints, with additional reference-assisted settings considered in their study. Second, although both studies explored UniProt-based partitioning on MdrDB, the exact preprocessing procedures, cross-validation folds, random seeds, and sample allocations are not identical. Third, the evaluation protocols differ because our study focuses on zero-reference structural inference, whereas prior work primarily examined sequence- or vector-based prediction and reference-assisted generalization.

Therefore, the purpose of this comparison is not to infer direct numerical superiority but to contextualize a shared overall trend: Both sequence-based and structure-aware paradigms show substantial performance degradation when evaluation shifts from conventional random splits to strict protein-disjoint UniProt-based splits.

More broadly, these findings align with recent studies showing that machine-learning benchmarks for biological macromolecules can be strongly influenced by data partitioning strategy and hidden train–test redundancy [48–50]. Similar concerns have also been raised in virtual screening and small-molecule property prediction, where models that perform well under conventional in-distribution splits often show substantial degradation under out-of-distribution (OOD) evaluation settings. For example, Fooladi et al. [51] systematically evaluated molecular property and activity prediction models across different OOD splitting strategies and highlighted that performance on random or in-distribution splits may not reliably reflect generalization to novel chemical space. This broader context reinforces our conclusion that a realistic split design is essential when assessing deployable models for mutation-induced drug resistance prediction.

### Differential mutation representations partially mitigate protein-background bias

To examine whether the ΔESM representation alters protein-identity structure in the embedding space, we performed a visualization analysis of the representational space formed by the WT embeddings and the ΔESM embeddings. Specifically, for each sample, the 1,152D ESM-C representation was standardized, reduced in dimensionality, and then projected into a 2D space using uniform manifold approximation and projection (UMAP) [52]. The resulting embedding distributions were colored according to UniProt ID.

**Fig. 6.**
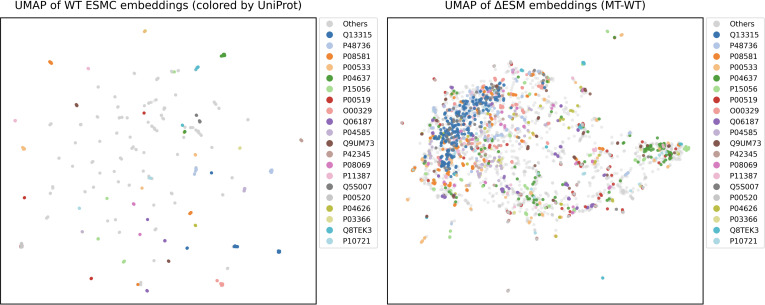
UMAP visualization of WT and ΔESM representations.

As shown in Fig. 6, the WT embeddings exhibit a pronounced protein-identity-driven clustering pattern in the representation space. Samples from the same UniProt cluster tightly together, while samples from different proteins form relatively distinct, separated groups. This indicates that the original WT representations retain substantial protein-identity-related information; that is, the embeddings encode, to a considerable extent, background features associated with which protein a given sample belongs to. Under the random split setting, such protein-level structure may provide the model with indirect identity cues, thereby improving predictive performance.

In contrast, the ΔESM embeddings display a markedly different geometric organization. Samples from different UniProt IDs are more intermixed in the representation space, and the protein-level clustering pattern is substantially attenuated. This suggests that the differential representation partially suppresses background information associated with protein identity, making the representation more focused on the relative perturbation signal induced by mutation rather than on the overall protein-specific context.

It should be emphasized that this visualization alone does not directly establish a causal explanation for the observed performance differences. However, from the perspective of representation-space geometry, it provides evidence in representation space that ΔESM reduces protein-background clustering.

To quantitatively support this visual observation, we computed the Silhouette score in the original 1,152D embedding space using UniProt ID as the grouping label. The WT embeddings yielded a Silhouette score of 0.256, consistent with a measurable protein-identity-driven clustering structure. In contrast, the score for the ΔESM embeddings decreased to −0.196, indicating that the differential representation substantially disrupts UniProt-level clustering. These results provide quantitative evidence that ΔESM mitigates protein-background identity bias and yields a representation space less dominated by global protein identity.

### Dataset noise and static-structure limitations constrain the current performance ceiling

To further understand the limited performance under the UniProt-based split, we examined both dataset-level noise and physical constraints imposed by the WT-complex-structure-only formulation.

1. Label noise and data heterogeneity. MdrDB integrates measurements from multiple literature sources and experimental conditions. As a result, a small number of identical protein–ligand–mutation records appear repeatedly but with discrepant ΔΔG annotations. Representative examples are shown in Table 6, and the complete list is provided in Table S6. These discrepancies likely reflect genuine experimental heterogeneity, including differences in assay protocol, temperature, pH, or related measurement conditions [53–55], and therefore represent an unavoidable source of observational noise in real-world ΔΔG data.

**Table 6.** Examples of duplicated protein–ligand–mutation entries in the MdrDB dataset that exhibit discrepant ΔΔG values across different literature sources

UniProt ID	Mutation	Drug	ΔΔG (source 1)	ΔΔG (source 2)	Abs. Diff
P00533 (EGFR)	L858R	Gefitinib	−4.04	−1.6	2.44
P00533 (EGFR)	G719S	Gefitinib	−1.53	0.48	2.01
P15056 (BRAF)	L597V	PLX-4720	−1.65	0.35	2.00
P15056 (BRAF)	A728V	PLX-4720	−2.51	−0.64	1.87

To quantify their impact, we performed an evaluation-level sensitivity analysis by removing all identified duplicate-conflict entries from the existing test predictions and recomputing the metrics without retraining. In total, 37 of 3,125 samples were removed, leaving 3,088 samples for filtered evaluation. The resulting changes were minimal: For both modes 0 and 3, the absolute change in pooled Pearson correlation was ≤0.01 and the change in MAE was ≤0.003 (Table S7). Thus, discrepant duplicate labels contribute to label noise, but they do not by themselves explain the performance collapse under the strict UniProt-based split.

2. Sparse coverage of protein space. The current dataset contains only a few thousand samples, and the UniProt-based split forces the training and test sets to contain disjoint protein targets. Given the diversity of protein sequences, structures, and ligand-binding mechanisms, this sparse coverage is insufficient to learn transferable physical rules across unseen protein backgrounds. The stronger performance under random splitting, therefore, reflects, at least in part, the benefit of shared protein backgrounds rather than robust out-of-protein generalization.

3. Physical limitations of static WT structures. ΔΔG reflects both enthalpic and entropic contributions [56]. Under the WT-complex-structure-only setting, the models can access only static WT structural geometry and mutation descriptors; they cannot directly observe mutation-induced conformational relaxation, altered side-chain packing, ligand-pose changes, or dynamics-related entropic effects. This limitation is particularly relevant for distal or allosteric resistance mutations, where the energetic effect may be mediated by conformational coupling rather than local static contacts [57].

Taken together, the performance ceiling observed in the strict UniProt-based setting likely arises from the combination of experimental label noise, sparse cross-protein data coverage, and missing conformational or dynamic information under the WT-only static-structure formulation.

## Conclusions

In this study, we assessed mutation-induced ΔΔG prediction in a practically relevant WT-complex-structure-only setting, where only the WT protein–ligand complex structure and known mutation annotation are available at inference time. Using the MdrDB CoreSet, we showed that model performance is highly sensitive to the data partitioning strategy. Under conventional random splits, models achieved a seemingly moderate predictive correlation, whereas performance declined sharply under strict UniProt-based evaluation. These results indicate that random splitting can substantially overestimate cross-protein generalization when predicting WT-only drug resistance.

Within this more realistic protein-disjoint setting, graph-based models that explicitly encode protein–ligand structural context retained weak but nonzero predictive correlation relative to vector baselines, suggesting that geometric information may provide limited complementary signals even when MT conformations are unavailable. Additional ablations further showed that reducing the graph to the ligand-proximal region and mutation-site neighborhood substantially degraded performance, whereas removing non-self message passing from the full-protein graph did not lead to a clear overall loss. These findings suggest that full-protein structural context is useful, but naive global message passing over static WT structures does not provide a complete solution; overall performance remained low, indicating that explicit structural context alone is insufficient for OOD ΔΔG prediction.

Taken together with our analyses of dataset characteristics and task formulation, the current performance bottleneck appears to arise from at least 3 factors: intrinsic experimental noise and heterogeneity in ΔΔG annotations, sparse coverage of protein space under strict cross-protein evaluation, and the absence of conformational and entropic information in the WT-only static-structure setting. These findings suggest that WT-only prediction should be treated as a distinct, more realistic evaluation scenario rather than a simplified variant of paired-structure prediction.

More broadly, we suggest that future studies should report both random-split and protein-disjoint results, explicitly quantify train–test overlap at the protein level, and avoid interpreting moderate random-split performance as evidence of robust real-world generalization. Future progress will likely require split-aware, protein-disjoint evaluation protocols, stronger physical priors, dynamics-related features, and broader high-quality cross-protein data coverage.

## Data Availability

The primary dataset used in this study is obtained from the Mutation-induced Drug Resistance Database (MdrDB), available at https://quantum.tencent.com/mdrdb/. All processed datasets, model implementations, and evaluation scripts are publicly available at https://github.com/LakiAo/WT-ddG-CriticalEval to ensure reproducibility. The pretrained protein language model ESM-C (600M parameters) used for feature extraction is available on Hugging Face at https://huggingface.co/EvolutionaryScale/esmc-600m-2024-12.

## Supplementary Materials

Supplementary 1 Sections S1 to S7 Tables S1 to S7
