# Supplementary material for: Beyond Random Splits: Assessing the Generalization of Graph and Vector Models for WT-Structure-Only Drug Resistance Prediction under Protein-Disjoint Evaluation
Source: Comput Struct Biotechnol J. 2026 Jun 18;35(1):0144. doi: 10.34133/csbj.0144 (PMC13276246; doi:10.34133/csbj.0144)
Supplement: Supplementary 1 — Sections S1 to S7 Tables S1 to S7 [file csbj.0144.f1.zip › Supplementary Material.docx]

# Supplementary Material

**Manuscript Title:** Beyond Random Splits: Assessing the Generalization of Graph and Vector Models for WT-Structure-Only Drug Resistance Prediction under Protein-Disjoint Evaluation

**Authors:** Zongrui Cheng, Haoxin Wu, and Dengming Ming

### Section S1. Detailed Protein and Sample Distributions Across Cross-Validation Folds

To provide transparent insight into the cross-protein evaluation landscape and illustrate the difficulty of the out-of-distribution evaluation, we quantified both the number of unique proteins, defined by distinct UniProt IDs, and the total sample counts for each fold.

The fold-by-fold numerical distributions are detailed in Table S1.

| Fold ID | Dataset Split | Number of Unique UniProt IDs | Total Sample Count |
| --- | --- | --- | --- |
| Fold 0 | Training | 106 | 1998 |
|  | Validation | 26 | 500 |
|  | Test | 34 | 627 |
| Fold 1 | Training | 106 | 2000 |
|  | Validation | 27 | 500 |
|  | Test | 33 | 625 |
| Fold 2 | Training | 106 | 1999 |
|  | Validation | 27 | 501 |
|  | Test | 33 | 625 |
| Fold 3 | Training | 106 | 2001 |
|  | Validation | 27 | 500 |
|  | Test | 33 | 624 |
| Fold 4 | Training | 106 | 2001 |
|  | Validation | 27 | 500 |
|  | Test | 33 | 624 |

**Table S1. Distribution of unique UniProt IDs and total samples per cross-validation fold under the strict UniProt-based split.**

### Section S2. Detailed model architecture and training hyperparameters

To improve reproducibility, Supplementary Table S2 summarizes the main architecture, graph-construction, optimization, and training hyperparameters used for the vector baseline and graph-based configurations. Mode 0 denotes the MLP-based vector baseline, whereas Modes 1–4 share the same heterogeneous graph backbone and training protocol but differ in residue-level input representation. Model-specific hyperparameters were fixed across all five cross-validation folds. Label normalization was computed using training-set statistics only and then applied to the validation and test sets.

| Category | Hyperparameter | Graph Model (Modes 1–4) | Vector Baseline (Mode 0) | Description / Note |
| --- | --- | --- | --- | --- |
| Architecture | Backbone | HeteroGNN (GINEConv) | MLP | - |
|  | Layers | 2 (Message Passing) | 3 (Linear Layers) | Depth of the learnable network |
|  | Hidden Dimension | 128 | 512 | Feature dimension in hidden layers |
|  | Dropout Rate | 0.35 | 0.25 | Applied after activation |
|  | Aggregation | Sum | - | Neighbor feature reduction strategy |
| Graph Construction | Protein Neighbors | k=16 | - | Based on Cα coordinates |
|  | Contact Threshold | 8.0 Å | - | Ligand-Protein interaction cutoff |
|  | Filter Threshold | 5.0 Å | - | Min dist. for valid complex filtering |
|  | Master Node | Enabled | - | Global context node |
|  | Self-loops | Enabled | - | Added to all nodes |
| Optimization | Optimizer | AdamW | AdamW | - |
|  | Learning Rate (LR) | 3.00E-04 | 6.00E-04 | Constant learning rate |
|  | Weight Decay | 5.00E-04 | 1.00E-04 | L2 regularization |
|  | Batch Size | 16 | 64 | - |
| Training | Loss Function | Huber Loss | Huber Loss | Robust regression loss |
|  | Label Norm. | Z-score | Z-score | Computed on training set only |
|  | Early Stopping | Patience = 20 | Patience = 20 | Based on validation performance |
|  | Random Seed | 42 | 42 | Fixed for reproducibility |
| Reproducibility | Param. Sharing | Yes | Yes | Model-specific settings were kept fixed across folds. |

**Supplementary Table S2. Detailed model architecture and training hyperparameters used for the graph-based configurations and vector baseline.**

Note: Modes 1–4 use the same graph backbone but differ in residue-level input features. Mode 0 denotes the vector baseline. Hyperparameters were fixed across folds for each model configuration. Label normalization was based only on the training set to avoid information leakage.

### Section S3. Hyperparameter Sensitivity Analysis and Ablation Study on Mode 3

To evaluate the sensitivity of the graph-based configuration used in the main analysis, we conducted a focused hyperparameter sensitivity analysis and ablation study for Mode 3 (Graph + One-hot + ΔESM) under the strict protein-disjoint UniProt split. The evaluated parameters included the k-nearest-neighbor (k-NN) density for intra-protein edges, the protein–ligand contact distance threshold, and the Gaussian radial basis function (RBF) width σ used in neighborhood pooling. In addition, we assessed the specific contribution of Gaussian neighborhood pooling by comparing it with standard mean pooling and configurations without neighborhood pooling.

The predictive performance across five-fold cross-validation, measured by the Pearson correlation coefficient (R) and Root Mean Square Error (RMSE), is summarized in Table S3.

| Hyperparameter | Setting / Value | Pearson R | RMSE (kcal/mol) |
| --- | --- | --- | --- |
| Neighborhood Pooling | Without Gaussian pooling | 0.1161±0.1110 | 1.2875±0.3799 |
|  | **Gaussian RBF pooling (Default)** | **0.1526±0.1481** | **1.2539±0.2749** |
|  | Mean pooling | 0.1250±0.1238 | 1.2729±0.3404 |
| Intra-protein Edges (k-NN) | 8 | 0.0926±0.1388 | 1.2943±0.3808 |
|  | 12 | 0.1091±0.0946 | 1.3015±0.3964 |
|  | **16 (Default)** | **0.1526±0.1481** | **1.2539±0.2749** |
|  | 24 | 0.0746±0.1263 | 1.2807±0.3295 |
|  | 32 | 0.0896±0.1299 | 1.2872±0.3926 |
| Contact Distance Threshold (Å) | 4 | 0.0273±0.1629 | 1.3323±0.3457 |
|  | 6 | 0.1186±0.0834 | 1.2770±0.3444 |
|  | **8 (Default)** | **0.1526±0.1481** | **1.2539±0.2749** |
|  | 10 | 0.1119±0.1419 | 1.2708±0.3715 |
|  | 12 | 0.0695±0.1358 | 1.3096±0.3655 |
| Gaussian Width (σ) | 1 | 0.0935±0.1277 | 1.2798±0.3824 |
|  | 1.5 | 0.1521±0.1665 | 1.2670±0.3920 |
|  | **2.5 (Default)** | **0.1526±0.1481** | **1.2539±0.2749** |
|  | 3.5 | 0.0856±0.1434 | 1.2748±0.3464 |
|  | 5 | 0.0864±0.1442 | 1.2972±0.3998 |

**Supplementary Table S3. Hyperparameter sensitivity analysis of the graph-based model under the strictly protein-disjoint UniProt split**

Note: Due to the inherent difficulty of the strict UniProt-based split, cross-protein ΔΔG prediction exhibits substantial variability across folds. Consequently, Pearson R remains within a low range across the tested configurations. These results support the reasonableness of the selected default parameters while also indicating that this task is sensitive to graph-construction choices.

### Section S4. Fold-Level Paired Statistical Analysis of Model Performance

To rigorously assess whether the numerical differences observed between Mode 3 and the other model configurations are statistically meaningful, we conducted a fold-level paired statistical analysis under the strictly protein-disjoint UniProt split. Given the substantial fold-to-fold variability inherent in out-of-distribution (OOD) evaluation, relying solely on aggregated means and standard deviations may make it difficult to assess whether the observed numerical differences are statistically supported. Therefore, we performed paired Wilcoxon signed-rank tests across the same five cross-validation folds used in our main evaluation.

The statistical comparisons were conducted between Mode 3 (Graph + One-hot + ΔESM) and the other four modes (Mode 0, 1, 2, and 4) across four key predictive metrics: Pearson correlation coefficient, Spearman correlation coefficient, Mean Absolute Error (MAE), and Root Mean Square Error (RMSE). To account for the multiple testing problem across different modes and metrics, all raw *p*-values were adjusted using the Benjamini–Hochberg False Discovery Rate (FDR) correction.

As summarized in **Supplementary Table S4**, while Mode 3 achieved the highest mean Pearson correlation—yielding an absolute mean difference of +0.1087 relative to the vector baseline (Mode 0)—this difference did not reach statistical significance (*p* = 0.3125, FDR-adjusted *p* = 1.0000). Furthermore, all other pairwise comparisons across Pearson, Spearman, MAE, and RMSE similarly yielded non-significant FDR-adjusted *p*-values. These statistical results indicate that the numerically higher Pearson correlation of Mode 3 should be interpreted as a limited trend rather than a statistically robust advantage. These findings further support a cautious interpretation of mode-level differences under the highly variable zero-reference cross-protein generalization setting.

| Metric | Comparison | Mean_Diff | Wilcoxon_p | FDR_p |
| --- | --- | --- | --- | --- |
| Pearson | Mode 3 vs Mode 0 | 0.1087 | 0.3125 | 1 |
| Pearson | Mode 3 vs Mode 1 | 0.0629 | 1 | 1 |
| Pearson | Mode 3 vs Mode 2 | 0.0196 | 0.8125 | 1 |
| Pearson | Mode 3 vs Mode 4 | 0.0493 | 1 | 1 |
| Spearman | Mode 3 vs Mode 0 | 0.0407 | 0.625 | 1 |
| Spearman | Mode 3 vs Mode 1 | -0.0595 | 0.4375 | 1 |
| Spearman | Mode 3 vs Mode 2 | -0.0392 | 1 | 1 |
| Spearman | Mode 3 vs Mode 4 | -0.0023 | 1 | 1 |
| MAE | Mode 3 vs Mode 0 | -0.0515 | 0.8125 | 1 |
| MAE | Mode 3 vs Mode 1 | 0.0245 | 0.625 | 1 |
| MAE | Mode 3 vs Mode 2 | 0.0344 | 0.3125 | 1 |
| MAE | Mode 3 vs Mode 4 | 0.0508 | 0.3125 | 1 |
| RMSE | Mode 3 vs Mode 0 | -0.0643 | 1 | 1 |
| RMSE | Mode 3 vs Mode 1 | -0.0119 | 0.8125 | 1 |
| RMSE | Mode 3 vs Mode 2 | 0.0161 | 0.625 | 1 |
| RMSE | Mode 3 vs Mode 4 | 0.0261 | 0.625 | 1 |

**Supplementary Table S4. Fold-level paired Wilcoxon tests comparing Mode 3 with other modes under the strict UniProt-based split.**

Note: Paired Wilcoxon signed-rank tests were performed across the same five cross-validation folds used in the main evaluation. FDR values were calculated using the Benjamini–Hochberg correction. A positive mean difference indicates a higher value for Mode 3 for the corresponding metric. For MAE and RMSE, lower values indicate better performance; therefore, a negative mean difference indicates lower error for Mode 3.

### Section S5. Target-level MAE stratification under the strict UniProt-based split

To assess target-dependent model behavior, we performed a target-level MAE stratification analysis under the strict UniProt-based split. Because PROTEIN_FAMILY annotations in MdrDB are fragmented and include many sparse or unknown entries, PROTEIN_NAME was used as the grouping variable. Major targets with at least 50 test samples were reported individually, whereas smaller targets were merged into “Other targets”.

For each target group, we reported the number of test samples, mean mutation–ligand distance, and MAE values for Mode 0 and Mode 3. ΔMAE was calculated as MAE_Mode0 − MAE_Mode3; therefore, positive values indicate lower MAE for Mode 3, whereas negative values indicate lower MAE for Mode 0.

| PROTEIN_NAME | N | Mean_Distance | MAE_Mode0 | MAE_Mode3 | Delta_MAE | Better |
| --- | --- | --- | --- | --- | --- | --- |
| Serine-protein kinase ATM | 219 | 56.90319 | 0.814931 | 1.063709 | -0.24878 | Mode 0 |
| Tyrosine-protein kinase ABL1 | 157 | 8.465361 | 0.570184 | 0.558767 | 0.011417 | Mode 3 |
| Phosphatidylinositol 4,5-bisphosphate 3-kinase catalytic subunit gamma isoform | 134 | 31.89183 | 1.020377 | 1.025189 | -0.00481 | Mode 0 |
| Hepatocyte growth factor receptor | 126 | 35.24745 | 0.578366 | 0.546922 | 0.031444 | Mode 3 |
| Epidermal growth factor receptor | 121 | 18.69771 | 1.039638 | 0.736824 | 0.302814 | Mode 3 |
| Cellular tumor antigen p53 | 119 | 18.30384 | 0.717854 | 0.592738 | 0.125116 | Mode 3 |
| Serine/threonine-protein kinase B-raf | 116 | 16.57288 | 0.931962 | 0.842142 | 0.089819 | Mode 3 |
| Phosphatidylinositol 4,5-bisphosphate 3-kinase catalytic subunit delta isoform | 115 | 31.97769 | 1.025625 | 1.108245 | -0.08262 | Mode 0 |
| Gag-Pol polyprotein | 115 | 11.07827 | 0.836021 | 0.897454 | -0.06143 | Mode 0 |
| Tyrosine-protein kinase BTK | 71 | 13.1523 | 0.94815 | 0.862182 | 0.085968 | Mode 3 |
| ALK tyrosine kinase receptor | 61 | 16.4655 | 0.924027 | 0.758593 | 0.165434 | Mode 3 |
| Serine/threonine-protein kinase mTOR | 59 | 56.18874 | 2.13458 | 2.275949 | -0.14137 | Mode 0 |
| Insulin-like growth factor 1 receptor | 55 | 29.0832 | 0.797635 | 0.648604 | 0.149031 | Mode 3 |
| DNA topoisomerase 1 | 53 | 27.2184 | 0.952924 | 0.902317 | 0.050607 | Mode 3 |
| Other targets | 1604 | 18.64286 | 1.026864 | 0.962325 | 0.064539 | Mode 3 |

**Supplementary Table S5. Target-level MAE stratification under the strict UniProt-based split.**

Note**:** Major targets with at least 50 test samples were reported individually. Mean_Distance denotes the average minimum mutation–ligand distance within each target group. ΔMAE = MAE_Mode0 − MAE_Mode3.

### Section S6. Duplicate-conflict entries with discrepant ΔΔG annotations

To document experimental label heterogeneity in MdrDB, we identified duplicate-conflict entries, defined as records with identical protein, ligand, and mutation annotations but discrepant experimental ΔΔG values. Because the complete list is large, it is provided as a separate supplementary data file: **Supplementary Table S6**.

These entries were retained in the primary dataset because discrepant annotations may reflect experimental heterogeneity across literature sources rather than simple data-entry errors. Their impact on evaluation metrics was quantified separately through the sensitivity analysis reported in Supplementary Table S7.

**Supplementary Table S6. Conflicting ΔΔG measurements for identical protein–ligand–mutation samples.**

Note**:** The complete duplicate-conflict entry list is provided as a separate supplementary data file. Duplicate-conflict entries were defined as records sharing the same protein, ligand, and mutation annotation but having discrepant ΔΔG values.

### Section S7. Sensitivity Analysis of Duplicate-Conflict Entries

As discussed in the main text, the MdrDB dataset contains a small fraction (~1.2%) of duplicate-conflict entries where identical protein-ligand-mutation triplets have discrepant experimental ΔΔG values. To quantify the impact of these discrepant annotations on evaluation metrics, we performed an evaluation-level sensitivity analysis on the test set predictions.

| Mode | N_orig | N_filt | Removed | Pearson_orig | Pearson_filtered | ΔPearson | MAE_orig | MAE_filt | ΔMAE |
| --- | --- | --- | --- | --- | --- | --- | --- | --- | --- |
| Mode 0 | 3125 | 3088 | 37 | 0.02 | 0.026 | 0.006 | 0.961 | 0.961 | 0 |
| Mode 3 | 3125 | 3088 | 37 | 0.077 | 0.075 | -0.003 | 0.923 | 0.926 | 0.003 |

**Supplementary Table S7. Sensitivity analysis of predictive performance after removing duplicate-conflict samples under the strict UniProt-based split.**

Metrics in this table were computed using pooled test predictions across all folds and are therefore not directly identical to the fold-wise mean ± SD values reported in the main tables. Duplicate-conflict entries were removed from the existing test predictions without retraining the models. Δ values denote filtered metric minus original metric.
